# Conventional and Innovative Processing of Milk for Yogurt Manufacture; Development of Texture and Flavor: A Review

**DOI:** 10.3390/foods3010176

**Published:** 2014-03-11

**Authors:** Panagiotis Sfakianakis, Constatnina Tzia

**Affiliations:** Laboratory of Food Chemistry and Technology, School of Chemical Engineering, National Technical University of Athens, 5 Iroon Polytechniou St., Polytechnioupoli, 15780, Zografou, Greece; E-Mail: psfakian@central.ntua.gr

**Keywords:** milk, yogurt, yogurt manufacture, thermal treatment, homogenization, pressure, ultra high pressure, ultrasound, microfluidization, pulsed electric fields, prebiotic, probiotic

## Abstract

Milk and yogurt are important elements of the human diet, due to their high nutritional value and their appealing sensory properties. During milk processing (homogenization, pasteurization) and further yogurt manufacture (fermentation) physicochemical changes occur that affect the flavor and texture of these products while the development of standardized processes contributes to the development of desirable textural and flavor characteristics. The processes that take place during milk processing and yogurt manufacture with conventional industrial methods, as well as with innovative methods currently proposed (ultra-high pressure, ultrasound, microfluidization, pulsed electric fields), and their effect on the texture and flavor of the final conventional or probiotic/prebiotic products will be presented in this review.

## 1. Introduction—History of Yogurt

Milk and dairy products have been consumed since the domestication of mammals; yogurt and similar fermented milk products in particular are thought to originate from the Middle East. The original production of fermented milk products derived from the need to prolong the shelf life of milk instead of being disposed [1]. Yogurt manufacture was initially based on knowledge and empirical processes without standard procedures or investigation of the steps that occur during the entire process. Only after the late 20th century, when yogurt became a profitable commercial good, its manufacture became industrialized and the processes were standardized. During the last 20 years, interest in yogurt manufacture has increased tremendously for scientific and commercial reasons. Scientific findings have suggested new dairy products that benefit human health (probiotic cultures, fortification with bioactive compounds) as well as with improved sensory, especially textural characteristics. Thus, consumer demand for yogurt and similar fermented dairy products has increased. 

Yogurt is defined as the product being manufactured from milk—with or without the addition of some natural derivative of milk, such as skim milk powder, whey concentrates, caseinates or cream—with a gel structure that results from the coagulation of the milk proteins, due to the lactic acid secreted by defined species of bacteria cultures. Furthermore, these bacteria must be “viable and abundant” at the time of consumption [2]. The above definition is part of the food legislation of many countries, ensuring that the essential characteristics of yogurt will be preserved, as well as that its traditional “concept” will not be compromised. The most common types of yogurt commercially available are set type yogurt and strained yogurt; though lately frozen yogurt and drinking yogurt have become quite popular as well. Set type yogurt is fermented in retail containers and no further stirring or water removal takes pace after the fermentation process. Strained (or stirred, or Greek style) yogurt is fermented in tanks under continuous mild stirring and after the completion of fermentation a portion of the whey is removed. Due to the manufacturing process, the two types develop a different texture; set type yogurt develops a continuous gel texture, whereas strained yogurt displays a viscous, creamy smooth texture [1].

## 2. Standardized Yogurt Manufacturing Process

Yogurt manufacture begins with the milking of the mammal, includes several processes, ending with the packaging of the final product, yogurt. The aim of this work is to present the processes that take place in the dairy industry; no further details about the milking and transportation of the milk will be included, even though these critical stages affect the quality and safety of the final product. Yogurt is mainly produced from bovine milk, although milk from other mammals is utilized for yogurt production as well. Yogurt derived from the milk of species other than bovine tends to vary in several sensory and physicochemical characteristics, due to differences per milk composition. For instance, yogurt derived from milk with high fat content (e.g., sheep, goat, and buffalo) has a more creamy texture compared to that derived from milk with lower fat content (e.g., bovine, mare, and ass). Therefore, the species of the milk-producing mammal significantly influence the characteristics of the produced yogurt [3].

### 2.1. Initial Treatment of Milk

Raw milk undergoes, in the dairy industry, centrifugal clarification to remove somatic cells and any other solid impurities [3]. Afterwards, a mild heating process, known as thermalization, is performed at temperature range 60–69 °C for 20–30 s, aiming at the killing of many vegetative microorganisms and the partial inactivation of some enzymes. This process causes almost no other irreversible change in the milk [4]. After thermalization, milk is cooled <5 °C or inoculated with lactic acid bacteria or other microfloras to control the growth of the psychrotrophic bacteria [3]. 

### 2.2. Standardization of Milk Components—Fat and SNF (Solid Non-Fat) Content

The standardization of milk refers to the standardization of fat and solid-non-fat content (SNF). Bovine milk fat content varies from 3.2%–4.2% w/w. The fat content of the milk is adjusted to range from <0.5%, for skim milk, to 1.5%–2%, for semi-fat milk, to 3.5% for full fat milk. As far as yogurt is concerned, the fat content ranges from 0.1%–10% according to consumer demands. In practice, to achieve the designed fat level, either the addition of skim milk or milk fat or the separation of fat from milk via centrifuge and mixing milk fat with skimmed milk is carried out [3]. The standardization process is of paramount importance, because the fat content of the milk influences the yogurt characteristics; increasing the fat content of milk results in an increase in the consistency and viscosity of yogurt [5,6]. Also, the milk fat content affects the maximum rate of pH decrease and pH lag phase during yogurt fermentation [7].

The term of standardization is also applied to the SNF content of the milk. The SNF components of milk mainly consist of lactose, protein and minerals; SNF content of milk varies from 11% to 14% of the total weight of the milk while the SNF of yogurt ranges from 9% to 16%. The SNF content of milk used for yogurt manufacture is altered, in some cases, by producers in order to attain the desired characteristics of the coagulum; the higher the SNF level, the higher the resulting yogurt’s viscosity and firmness. The addition of native milk components is permitted to yogurt and fermented milk products in some countries. It is quite common in yogurt manufacturing to fortify the milk mixture with milk powder (skimmed or full fat), whey protein concentrates or casein powder, to achieve the desired SNF content and subsequently an increase in firmness and cohesiveness [5]. It must be noted that the fat and SNF content of milk has an impact on the fermentation process. In particular, the interaction of milk SNF content and fermentation temperature has a significant effect on the duration of the fermentation process; an increase of SNF increases the duration of the fermentation process [8].

### 2.3. Homogenization

Milk is a typical oil in water (o/w) emulsion with milk fat globules (MFG) acting as the oil droplets and the milk fat globules membrane as the emulsifier. However, because of the reaction of agglutinins and interfacial tension, the fat globules tend to collide, either by sharing the membrane or because of the Laplace principle, according to which the pressure is greater inside small globules than inside large globules and, hence, there is a tendency for large fat globules to grow at the expense of the smallest. This phenomenon, in addition to Brownian motion, forces the milk fat to rise to the surface of the milk and thus creates the undesirable effect of separation [9,10]. In order to prevent this effect, standardized milk undergoes homogenization. The basic principle of milk homogenization is to subject MFG to severe conditions in order to disrupt the membrane surrounding them and then maintain the new globules in dispersion while a new membrane is formed at the fat serum interface. The severe conditions that cause milk homogenization can be achieved by the application of pressure, high velocity flow of the milk, or high frequency vibrations (>10 kHz). The shear stress and temperature gradient developed under these conditions lead to a cavitation phenomenon that contributes to the homogenization process. Homogenization is carried out by the application of pressure. In particular, the pressure commonly applied in the dairy industry is 10–20 MPa [11,12]. The main homogenization effects are a reduction of the diameter of the MFG from 2–10 μm to 0.1–1 μm and altering the composition of the MFG membrane. According to Cano-Ruiz and Richter [13], the membrane absorbs protein molecules, mostly caseins, from the milk serum to become sufficient to emulsify the new-formed globules, since the fat surface area increases due to homogenization. Aguilera and Kessler [14] showed that a reduction of MFG size and the alterations in the MFG membrane caused by homogenization contribute to milk emulsion stability. Furthermore, homogenization affects the characteristics of acidified milk gels, like yogurt. According to Cho, *et al.* [15], the smaller MFG facilitate the incorporation of fat into the protein network [5], while their increased surface area favors the interactions between fat and milk proteins, casein and denatured whey, during acidification and subsequent gel formation [5,15].

### 2.4. Heat Treatment

Heat treatment of milk is carried out to ensure the safety of the product, whether it is milk itself or any other dairy product, and to exploit several effects that increased temperature has on certain milk components facilitating further processes for dairy products manufacture [16]. Heat treatment of milk reduces the number of pathogenic microorganisms to safe limits for the consumer’s health. Various heat treatments can be applied, which are classified based on the duration and the temperature (Table 1). The most common are known as thermalization (referred in Section 2.1), low and high pasteurization, sterilization and UHT (Ultra Heat Treatment) [3,4,17]. Low pasteurization refers to heat treatment of milk at 63–65 °C for 20 min or at 72–75 °C for 15–20 s (HTST, High Temperature Short Time). During this process, most pathogens, vegetative bacteria, yeast and molds are killed. Additionally, with low temperature pasteurization, several enzymes become inactive, while the flavor of milk is hardly altered. Furthermore, little or no serum proteins are denatured, and cold agglutination and bacteriostatic properties remain virtually intact [2,4]. A more intense heat treatment is high temperature pasteurization that requires a temperature of 85 °C for 20–30 min or 90–95 °C for 5 min. During high temperature pasteurization most vegetative microorganisms are killed, except from spores; most enzymes are deactivated (except milk proteinase, plasmin in particular, some bacterial proteinases and lipases); most whey proteins are denatured, and a distinct “cooked” flavor is developed due to the formation, mostly, of ketones [4,18]; no further irreversible changes occur. Sterilization results in extermination of all microbial content of milk, including bacterial spores, and it is achieved at 110 °C for 30 min or at 130 °C for 40 s. In addition, sterilization causes inactivation of most milk enzymes (except several bacterial lipases), darkening of the milk color due to the Maillard reaction, evaporation of most flavor volatiles, thus weakening the flavor of the milk, and considerable damage to all milk proteins, even caseins. Finally, UHT is carried out at 145 °C for 1–2 s and achieves equal bacterial eradication as from sterilization, minimal flavor deterioration and causes denaturation of several whey proteins (β-lactoglobulin, serum albumin, and some immunoglobulins). UHT treatment and high pasteurization produces many volatiles in milk, such as: 2-pentanone, 2-heptanone, 2-nonanone, 2-undecanone, 2,6-dimethylpyrazine, 2-ethylpyrazine, 2-ethyl-3-methylpyrazine, methional, pentanoic acid, benzothiazole vanillin, hexanal, benzothiazole, decalactone, H_2_S, methanethiol, dimethylsulphide and carboxylsulphide. These sulfur containing molecules are responsible for the “cooked” off flavor developed during UHT and high temperature pasteurization [18]. It should be mentioned that the most commonly used heat treatment in the yogurt manufacturing process is the high temperature pasteurization at 85 °C for 20 min [3,4].

Pathogens that can grow in milk, due to bad hygiene practices or hardware failure during the stages of processing, include *Mycobacterium tuberculosis*, *Coxiella burnetii*, *Staphylococcusaureus*, *Salmonella* species, *Listeria monocytogenes*, and *Campylobacter jejuni*. These microorganisms are killed by even mild heat treatment ensuring that processed milk is safe for consumption. The claim that milk is safe after a mild heat treatment might sound frivolous, but most high-heat resistant pathogens either do not occur in milk (e.g., *Bacillus anthracis*) or are outnumbered by other native microorganisms (e.g., *Clostridium perfringens*), or cause spoilage before their quantity is enough to cause health issues (e.g., *Bacillus cereus*).

In addition to the reduction or complete extermination of microbiological load, heat treatment causes release of CO_2_ and O_2_, an increase in the amount of insoluble colloidal calcium phosphate, a decrease in calcium cations, and forces lactose isomerization, degradation and Maillard reaction, thus affecting the pH of the milk and flavor. Finally for yogurt, the most important changes during heat treatment of milk concern the milk proteins; the reactions of milk proteins, during heat treatment, have a serious impact on the yogurt curd formation and will be described more thoroughly [4].

The casein molecules in milk are in the form of micelles or aggregates of submicelles which are formed from α_s1_-, α_s2_- and β-caseins stabilized by κ-casein molecules held together by calcium and calcium phosphate. This structure is stable and requires a high amount of energy to be disrupted. On the other hand, whey proteins in solution have a globular shape. Whey proteins, due to their structure, are fairly stable and do not interact with fatty molecules, calcium ions or caseins in their native state. However, in the case of whey proteins (β-lactoglobulin, serum albumin) over 80 °C, their peptide chains unfold, thus denaturating irreversibly. This deformation of the peptide chains exposes their thiol groups and enables them to interact with other molecules forming S–S bonds. Depending on the pH of the environment and the proximity of molecules available, whey proteins can form bonds with other whey proteins and caseins (κ- and α_s1_- mostly) and also they can be incorporated at the MFG membrane. The denatured whey proteins, especially at pH values lower than 6.5, have the tendency to associate with casein micelles [4,11,16]. All the above phenomena are of paramount importance and are exploited during yogurt manufacture. Yogurt curd formation is based on the isoelectric precipitation of casein. However, whey proteins can be involved; if the thiol groups of the whey proteins are exposed, an interaction between casein and whey protein molecules occur, and the formation of casein-whey bonds are facilitated. Thus, whey proteins are incorporated into the curd matrix, strengthening the latter and resulting in a more firm yogurt. Therefore, the heat induced denaturation of whey protein favors the yogurt formation with high firmness and viscosity values [5,19]. Table 1 summarizes the thermal treatments utilized in dairy processing and the respective effects on milk itself and yogurt.

**Table 1:** Impact of different thermal treatment techniques on milk and yogurt properties affecting flavor and texture.

Milk Treatment	Treatment Description	Effect on Milk	Effect on Yogurt
Thermalisation	Heating at 60–69 °C, for 20–30 s	Death of non-heat resistance bacteria.	No significant effect.
		Inactivation of several enzymes [4].	Characteristics affected by further processing [4].
Low Pasteurization	Heating at 63–65 °C for 20 min/at 72–75 °C for 15–20 s (HTST)	Death of most pathogens, vegetative bacteria, yeast and molds.	Slight increase in viscosity and firmness [1].
		Several enzymes denatured, denaturation of several whey proteins [4].	
High Pasteurization	Heating at 85 °C for 20–30 min/at 90–95 °C for 5 min	Death of most vegetative microorganisms, except spores.	Large increase in viscosity and firmness [1].
		Deactivation of most enzymes.	
		Denaturation of most whey proteins.	
		Development of “cooked” flavor [4,18].	
Sterilization	Heating at 110 °C for 30 min/at 130 °C for 40 s	Extermination of all microorganisms.	Incorporation of whey proteins into casein matrix. Very large increase in viscosity and firmness [1,4].
		Deactivation of most enzymes.	
		Denaturation of whey proteins and aggregation of caseins (casein micelles) and MFG.	
		Weakening of flavor intensity.	
		Color darkening [4,18].	
Ultra Heat Treatment (UHT)	Heating at 145 °C for 1–2 s	Extermination of all microorganisms.	Medium increase in viscosity and firmness [1].
		Mild flavor deterioration.	
		Denaturation of whey proteins (β-lactoglobulin, serum albumin, several immunoglobulins)	
		Development of off-flavors.	
		Color darkening [4,18].	

### 2.5. Fermentation Process

The fermentation process is the most important stage of yogurt manufacture. During this stage, the yogurt curd is formed, and its textural characteristics and distinct flavor are developed [3,5]. The key factor of the fermentation process is the starter culture that acts through biochemical reactions and inductively causes the formation of the curd and the development of flavor components [5]. For a fermented dairy product to be labeled as “yogurt”, it should contain the two live bacterial strains of *Streptococcus salivarius* subsp. *thermophilus* and *Lactobacillus delbrueckii* subsp. *bulgaricus* in abundance. However, yogurt starter cultures may include other microorganisms as well, like *Lactobacillus acidophilus*, *Lactobacillus casei*, *Lactobacillus lactis*, *Lactobacillus jugurti*, *Lactobacillus helveticus*, *Bifidobacterium longum*, *Bifidobacterium bifidus* and *Bifidobacterium infantis*. *Streptococcus thermophilus* subsp. *thermophilus* (ST) is the only species in the streptococcus genus that is used in dairy starter cultures. ST is Gram positive and usually considered thermophilic, however, as the optimum temperature for its growth is 35–53 °C; therefore, ST can be considered as “thermotolerant”. Its cells are spherical in shape, forming chains, during the early stage of their lives and as they mature develop a more rod-like morphology and favor colonial growth. *Lactobacillus delbrueckii* subsp. *bulgaricus* (LB) is rod-shaped, Gram-positive, anaerobic bacteria and its optimum growth temperature is 40–44 °C. LB can produce very high amounts of lactic acid by metabolizing lactose [5,20]. These two species display synergy in the milk environment, metabolizing lactose into lactic acid and causing reduction of milk pH. The synergism between ST and LB is based on their individual characteristics, and as a result higher lactose metabolism and lactic acid production is attained compared to each one acting individually. ST is more “aerotolerant” than LB, lacks good proteolytic ability in comparison to LB, but possesses greater peptidase activity. When grown together in milk, ST grows vigorously at first, whereas LB grows slowly. ST, because of its great proteolytic activity, creates an abundance of peptides to stimulate the growth of LB. During the early stage of fermentation, milk lactose is transported through the cell membrane of ST with the help of the enzyme galactoside permease located in the membrane. The lactose in the cell is then hydrolyzed by lactase or β-galactosidase enzyme. ST produces significant levels of lactase, which catalyzes the hydrolysis of lactose to glucose and galactose. Glucose is converted to pyruvate which is metabolized to lactic acid by the enzyme lactic dehydrogenase. Lowered oxygen tension and formate (byproduct of ST metabolism) in turn stimulate LB growth, which is further aided by the amino acids released by the active peptidases secreted by ST. Through coordinated tandem activities, both bacteria accelerate the entire fermentation, which none of them would be able to achieve individually. When the pH of the yogurt approaches 5.0, activity of ST subsides and LB gradually dominates the overall fermentation process until the target value of pH is reached and the fermentation process ceases. Normally, the fermentation period is terminated by lowering the temperature to 4 °C. At this temperature, the culture is still alive, but its activity is drastically limited to allow controlled flavor during storage and distribution [5,20].

The growth of the symbiotic culture induces changes in the native components of the milk that are responsible for the physicochemical and sensory characteristics of yogurt. During fermentation, lactose, milk proteins and microbial content, as well as several carbon compounds, suffer major changes, whereas minor changes occur for vitamins and minerals. Lactose is reduced by 30% and produces double the molar amount of lactic acid. Proteins (caseins and whey) aggregate, increasing the consistency of yogurt. Due to proteolysis caused by starter culture, amino acids (mainly proline and glycine) are released into the yogurt, even during storage at 4 °C [20]. During incubation, the starter culture growth results in an increase in the system’s microbial content from 10^8^ to 10^10^ CFU g^−1^. The carbonyl compounds formed during fermentation are mostly lactic acid, acetaldehyde, dimethyl sulfide, 2,3-butanedione, 2,3-pentanedione, 2-methylthiophene, 3-methyl-2-butenal, 1-octen-3-one, dimethyl trisulfide, 1-nonen-3-one, acetic acid, methional, (*cis*,*cis*)-nonenal, 2-methyl tetrahydrothiophen-3-one, 2-phenyacetaldehyde, 3-methylbutyric acid, caproic acid, guaiacol and benzothiozole. These compounds contribute to the distinctive flavor of yogurt. As far as the lipids are concerned, several free fatty acid molecules are liberated due to lipase activity, mostly stearic and oleic acid. The only change in vitamin content is an increase in Vitamin B, throughout the fermentation process and storage. Finally, the quantity of minerals in yogurt remain the same as in milk, the only change is that, due to pH lowering, these minerals are in ionic rather than colloidal form [5,18].

The concentration of lactic acid in milk during fermentation increases, pH decreases, therefore the carboxyl groups dissociate, serine phosphate is ionized, and the negative charge between casein micelles is increased. However, the presence of calcium phosphate neutralizes this negative charge, keeping electrostatic repulsion down to a level where attractive forces between the protein molecules are dominant. Due to these attractive forces, the casein micelles aggregate and eventually coagulate into a network of small chains; this is responsible for the increase of viscosity and formation of the yogurt coagulum [3,19,21,22].

The milk fermentation process of yogurt can be described adequately by the evolution of pH and viscosity with respect to time; the model that expresses the evolution of pH during fermentation time is the modified Gompertz models of de Brabandere and de Baerdemaeker (1999) (Equation (1)) [23]:

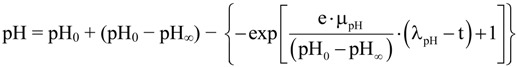
(1)
pH_0_, pH_∞_ = initial and end values of pH, respectively; μ_pH_ (min^−1^) = maximum rate of pH decrease; λ_pH_ (min) = duration of pH lag phase. Furthermore, the model that describes the evolution of viscosity during fermentation is the modified Gomperz model of Soukoulis, *et al.* (2007) (Equation (2)) [7]:

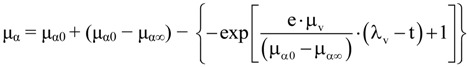
(2)
μ_α0_, μ_α∞_ (Pa·s) = initial and end values of viscosity respectively; μ_v_ (min^−1^) = maximum rate of viscosity decrease; λ_v_ (min) = duration of viscosity lag phase.

### 2.6. Cooling

After the pH of yogurt reaches the value of 4.7–4.3, the yogurt is cooled to around 5 °C. This inhibits the growth and metabolic reaction of the starter culture and prevents the rise in acidity. Cooling of yogurt can be in one or two phases. One-phase cooling involves the rapid decrease of the coagulum temperature to less than 10 °C, where the fermentation process is inhibited leading to yogurt with low viscosity. Two-phase cooling is initiated by rapidly decreasing the temperature to less than 20 °C and then gradually reaching the storage temperature of 5 °C leading to yogurt with an increased viscosity and limited syneresis. This is quite common in the yogurt manufacture process, especially when fruits are to be added [3,24].

## 3. Innovative Methods for Milk and Yogurt Processing

Among the processes involved in milk and yogurt processing, the most important are homogenization, pasteurization and fermentation. Apart from conventional processes previously mentioned, new trends in milk processing that involve the utilization of ultra high pressure, ultrasound, pulsed electric field and microfluidization are presented in this section. Last, but not least, the probiotic bacteria and prebiotics are discussed that have beneficial effects on human health and have extensively been utilized in the dairy industry. Probiotics and prebiotics are worth mentioning, as dairy products are the most common medium for delivering them to the human intestine. In Table 2, the effects of conventional or innovative methods of homogenization on milk and yogurt properties are presented that generate the flavor and texture characteristics.

### 3.1. Ultra High Pressure Milk Treatment and Effect on Yogurt Characteristics

Ultra high pressure (UHP) involves the application of pressures from 100 to 1000 MPa. UHP utilization in food products was initiated during the early 1980s and is a non thermal pasteurization method. Studies have shown that milk treatment with pressures of 400–600 MPa for 10 min at 25 °C can achieve a similar result to low temperature pasteurization in terms of pathogenic and spoilage microorganisms inactivation [25]. Similar studies on UHP treatment of milk from Johnston, *et al.* [26] and Law, *et al.* [27] described the disintegration of the casein micelles into smaller particles and the simultaneous increase in the amount of caseins and calcium phosphate in the serum phase. Also, it was found by the same researchers that after UHP treatment, especially with pressure higher than 500 MPa, that the denaturation of several whey proteins occurs, in particular β-lactoglobulin, several immunoglobulins and α-lactalbumin [25,28]. However, Gervilla, *et al.* [29] observed a strange effect of UHP on the size and distribution of MFG. UHP up to 500 MPa at 25 and 50 °C reduced the diameter of MFG in the range of 1–2 μm, but at 4 °C the MFG displayed no tendency to shrink. This could be attributed to the fact that the MFG membrane remained unchanged.

The application of UHP in milk acid gel formation improves the texture and firmness, reduces syneresis and increases the water holding capacity in comparison to conventional yogurts [25]. The combination of UHP and thermal treatment is reported by Ferragut, *et al.* [30] to increase yogurt viscosity and lower gelation times compared to UHP treated samples.

### 3.2. Ultrasound Milk Treatment and Yogurt Characteristics

Ultrasound (US) is a sound wave with a frequency higher than the upper limit of human hearing, typically higher than 20 kHz. US has been utilized in the food industry since the late 1960s, for cleaning, monitoring and food component characterization. High intensity US (power level higher than 10 W), when propagated through a solution, generate immense pressure, temperature and shear gradients and thus cause cavitation [31,32]. Therefore, US is considered as an alternative method for reducing MFG size and can be effectively applied to homogenize milk. Wu, *et al.* [33] and Nguyen and Anema [34] showed that the application of US in milk reduces the MFG diameter to between 0.1 and 0.6 μm. In addition, US treatment has been referred by Krešić, *et al.* [35] and Chandrapala, *et al.* [36] to cause alterations of the MFG membrane composition and structure leading to an efficient homogenization effect compared to conventional methods. Additionally, the effect of US on milk proteins has been studied by Madadlou, *et al.* [37] and by Chandrapala, *et al.* [36]. US treatment has been shown to cause alteration in the secondary structure of the milk proteins, aggregation of protein particles as well as denaturation [36,37]. Riener, *et al.* [38] combined US with heat treatment (thermosonication) of milk and achieved a similar effect on the MFG as obtained with US treatment without heat, leading to reduction in size and changes of the membrane allowing interaction with casein micelles. Specifically, thermosonication treatment leads to an average diameter of 0.6 μm of MFG and a MFG membrane richer in casein molecules than the native [38]. Furthermore, high amplitude US has been reported to reduce the microbial content of milk [32,39]. Finally, high intensity US treatment causes the emission of volatiles from milk and formation of off flavors. Based on the study conducted by Riener, *et al.* [40], when milk is ultrasonicated, benzene, toluene, 1,3-butadiene, 5-methyl-1,3-cyclopentadiene, 1-hexene, 1-octene, 1-nonene, p-xylene, *n*-hexanal, *n*-heptanal, 2-butanone, acetone, dimethylsulfide and chloroform are emitted. The aldehydes can be produced from the breakdown of hydroperoxides generated by photo-oxidation induced by US, whereas the series of C6–C9 1-alkenes could arise from pyrolytic cleavage of fatty acid chains. The benzene formation may be attributed to cleavage of side chains of amino acids such as phenylalanine. These volatiles cause a rubbery and burned aroma [40].

The implementation of US treatment on the production of fermented dairy products has been studied with promising results. Milk gels and yogurt produced from milk treated by high intensity US have shown improved physical properties and high value of texture characteristics (firmness, cohesiveness). Increased amplitude level US treatment (20 kHz, 50–500 W, 1–10 min) significantly improved the water holding capacity of yogurt and increased viscosity and reduced syneresis; moreover, higher US intensity and a higher US exposure time of the milk resulted in increased yogurt viscosity [33]. Increased yogurt viscosity was reported by Riener, *et al.* [38] even from skim milk treated with US (22 kHz, 50 W, 0–30 min), due to high thermal denaturation of whey proteins. Milk thermosonication prior to fermentation, (25 kHz, 400 W, 45 or 75 °C for 10 min) resulted in the formation of yogurt with greater viscosity and higher water holding capacities compared to conventionally treated milk. The same treatment altered the microstructure of yogurt resulting in a honeycomb like network and exhibiting a more porous nature, whose average structural size was smaller (~2 μm) compared to conventionally heated yogurt [40]. A study by Vercet, *et al.* [41] combined thermosonication treatment of milk (40 °C, 20 kHz for 12 s) with moderate pressure (2 kg × cm^−2^) and showed that the apparent viscosity, yield stress, and viscoelastic properties of yogurt were increased in addition to its structure being strengthened. The increased viscosity and texture of yogurt derived from ultrasonicated milk can be attributed to the denaturation of whey proteins and association of the latter with caseins. Denatured whey proteins are more susceptible to association with casein and casein micelles. Additionally, during acidification the denatured whey proteins, associated or not with casein micelles, aggregate due to the reduction of the repulsive charge. Therefore, denatured whey proteins associated with casein micelles could act as bridging material between casein micelles and as a result the bonds that form in the yogurt matrix are formed more easily, resulting in stronger yogurt coagulum [42].

### 3.3. Application of Microfluidization in Milk and Yogurt Manufacture

The Microfluidizer^®^ is an apparatus that causes homogenization via shear, turbulence and cavitation. Initially, it accelerates the fluid and separates it into two microstreams that intersects in a chamber and collide. The impact causes intense turbulence and cavitation and thus the homogenization effect is achieved [43,44]. In the case of milk studied by Ciron, *et al.* [43], the microfluidization treatment reduced the diameter of the MFG to less than 2 μm [45]. The application of microfluidization in yogurt manufacture has often been used. A comparison between yogurts derived from microfluidized milk of 0% and 1.5% fat content with conventionally homogenized milk showed that non-fat yogurt from microfluidized milk displayed increased syneresis, and reduced viscosity and firmness compared to conventionally manufactured yogurt, whereas low fat yogurts from microfluidized milk had similar texture characteristics with those from conventionally homogenized milk. Microfluidization of low-fat milk resulted in yogurt with modified microstructure, giving more interconnectivity in the protein networks with embedded fat globules, but with similar texture profiles and water retention compared to yogurt prepared from conventionally homogenized milk [43]. However, this technique requires more study to assess the efficiency of yogurt manufacture [43,46].

### 3.4. Pulsed Electric Field Application in Milk and Yogurt Manufacture

Pulsed electric field (PEF) treatment emits intense electric pulses through a continuous medium, to inactivate microorganisms with the best results achieved in fluids. PEF has been applied in dairy systems combined with probiotic cultures [47]. The intensity of fields range between 15 and 50 kV/cm, and the treatment lasts only a few seconds [48]. The PEF principle is to destabilize the microbial cells with a high-pressure pulse. Subsequently, electroporation to the cellular membrane makes it more permeable; therefore, the cells rupture and expel their contents [49]. Additionally, Lin, *et al.* [50] combined PEF, UHP and thermal treatment in milk and noticed even more of a decrease in microbiological content. The efficiency of PEF depends on the intensity of the electric field, and the number and duration of pulses [51]. Despite its potential, PEF application requires a high tolerance to elevated electric fields, low electric conductivity and absence of bubbles [51,52].

**Table 2:** Impact of different homogenization techniques on milk and yogurt properties affecting flavor and texture.

Milk Treatment	Treatment Description	Effect on Milk	Effect on Yogurt
Conventional with Pressure	10–20 MPa	Decrease of MFG size.	Facilitation of curd formation. Whey protein incorporation into the casein matrix. Slight increase in viscosity and firmness.
		Stability of milk as an emulsion.	
		Whey proteins absorption to the MFG membrane.	
Ultra High Pressure (UHP)	100–1000 MPa	Inactivation of spoilage and pathogenic microorganism.	Higher value of texture characteristics. Higher viscosity. Lower syneresis. Increased water holding capacity.
		Casein micelles disruption.	
		Denaturation of several whey proteins.	
		MFG size decrease with a tendency for collision and re-aggregation.	
High Intensity Ultrasonication	Higher than 20 kHz, amplitude higher than 100 W	MFG size reduction.	Higher value of texture characteristics. Higher viscosity. Lower syneresis. Increased water holding capacity.
		Stability of milk as an emulsion.	
		Interaction of whey proteins with casein micelles and MFG.	
		Reduction of microbial content.	
		Development of off-flavor volatiles.	
Microfluidization	Separation of milk into two steams, moving at high velocity with subsequent collision.	MFG size reduction.	Non-fat yogurt: increased syneresis and lower viscosity.
			Low fat yogurt: similar texture characteristics as for conventionally manufactured yogurt.
Pulsed Electric Field (PEF)	Application of electric pulses through milk.	Microbial content reduction.	Similar texture and water holding capacity as for conventionally manufactured yogurt.
	Intensity: 1–50 kV/cm for a few seconds.		

### 3.5. Probiotic and Dairy Products

Modern nutritional probiotic products are classified as those that contain probiotic microorganisms. Probiotic microorganisms are defined as live microbes, which when ingested benefit the health of the host through their effect on the intestinal microflora. In addition, probiotic cultures must be able to survive throughout the intestinal tract, resist acidic conditions during gastric passage and bile digestion. To have their beneficial effect on the health of the host, probiotic strains must, at least temporarily, establish themselves among the natural microflora of the intestine. The initial mention about microorganisms that benefit the health of host being, while delivered via food consumption is attributed to Élie Metchnikoff in the early years of the 20th century [53]. The terminology “probiotic” is attributed to Lilley and Stillwell [54] in order to differentiate them from antibiotics. These authors defined probiotics as a substance produced by one microorganism stimulating the growth of another microorganism [54]. Later on, in 1971, Sperti gave this name to tissue extracts promoting the growth of microbes [55]. The definition by Parker in 1974 is closer to the commonly accepted definition: organisms and substances which contribute to the intestinal microbial balance [56]. The accepted definition of probiotics is a slightly improved definition by Fuller in 1989 “live microbial feed supplement which beneficially affects the host by improving its intestinal microbial balance” [57]. Probiotics are added to food as adjunct cultures in concentrations of 10^7^–10^8^ CFU/g or mL, if not participating in the fermentative process, and if participating, they can reach a concentration between 10^8^ and 10^9^ CFU/g or mL after the fermentative process [58]. The most common probiotic bacteria are strains and species of Lactobacilli, Bifidobacteria, Enterococci and Lactococci. The species most thoroughly been studied are *Lactobacillus acidophilus*, *Lactobacillus casei*, *Lactobacillus lactis*, *Lactobacillus helveticus*, *Bifidobacterium longum*, *Bifidobacterium lactis*, *Bifidobacterium animalis* ssp. *lactis* and *Bifidobacterium bifidum*, *Bifidobacterium longum*, and *Bifidobacterium bifidus* [59].

Fermented dairy products, and especially yogurt, are ideal carriers for probiotic cultures to enter the human digestive system and ensure their survivability through the stomach [60]; therefore, the subject of probiotic yogurt is thoroughly studied from its medical and dairy science perspective. For the purpose of this work, only the effect of the probiotic cultures on the yogurt manufacture and characteristics is mentioned.

Most probiotic bacteria have no significant effect on the fermentation process or on the yogurt sensory properties [61]. Based on the studies of Allgeyer, *et al.* [61] and Atunes, *et al.* [62], the addition of *Bifidobacterium lactis* and *Lactobacillus acidophilus* resulted in no difference in the sensory evaluation of low and full fat probiotic and non-probiotic yogurt. The same results were noted by other researchers as well [63,64]. However, according to Akalin, *et al.* [65] the combination of probiotic culture (*Bifidobacterium animalis* ssp. *Lactis*) and fortification of WPC (whey protein concentrate) resulted in a stronger coagulum and increased firmness and adhesiveness values for the yogurt. This was attributed mostly to the effect of the fortification with WPC and not to the presence of the probiotic culture.

Overall, the incorporation of probiotic bacteria in yogurt manufacture is viable and this claim is shown by the amount of probiotic yogurt products available in the market; however, the actual challenge is to ensure that the probiotic culture reaches the intestines of the consumers alive and is able to establish itself among the native microflora [66].

### 3.6. Prebiotics and Dairy Products

According to Gibson and Roberfroid [67], prebiotics are classified as certain food ingredients that beneficially affect the host in a very specific way. Prebiotics are food components, non-digestible by humans, that selectively stimulate the growth and activity of certain bacterial species already existing in the human colon, and inductively improve the health of the host. Most prebiotics are oligosaccharides in general, fructooligosaccharides in particular. Most common oligosaccharides with prebiotic character are inulin, *trans*-galactooligosaccharide, lactulose, isomalt and oligofructose. They have been shown to stimulate the growth of endogenous Bifidobacteria, and make them the predominant species in human feces [68]. Based on the study of Cruza, *et al.* [69] who added oligofructose to plain yogurt, there was no influence on the pH, the proteolytic ability, or on the viability of *Streptococcus thermophilus* or *Lactobacillus bulgaricus*. However, the end product of this endeavor was characterized as a weak gel with thixotropic and pseudoplastic behavior. Finally, the oligofructose-fortified yogurts had a fairly high acceptance by consumers. Another study conducted by Pimentel [70] suggested that the addition of long chain inulin, another known prebiotic, in low fat yogurt can lead to interesting results in sensory properties and especially in texture characteristics. In particular, the replacement of native milk fats with long chain inulin created equally acceptable firmness and color as with yogurt containing native milk fats. Most studies on the addition of prebiotic oligosaccharides in yogurt agree that most of the characteristics of the final product and the process remain fairly close to the values of the originals. Several short chain prebiotics have a slightly negative effect on the firmness and creaminess of the yogurt whereas long chain prebiotics increase those values. Overall, the final choice remains with the consumer and their preference for texture.

## 4. Conclusions

Dairy processing and yogurt manufacture utilize several scientifically interesting procedures such as centrifugation, homogenization, heat treatment, and in the case of yogurt, manufacture and fermentation. Each procedure significantly affects the quality and sensory characteristics of the final product, whether it is milk or yogurt. Conventional heat treatment includes thermalization, low and high temperature pasteurization, ultra heat treatment and sterilization. The application of heat treatment in milk affects the flavor, the microbial content and the milk proteins. The more intense the heat treatment is, the more radical the changes that occur. Heat treatment also affects the texture of the produced yogurt, increasing the value of its texture characteristics (firmness, cohesiveness) and viscosity. Homogenization, typically used in dairy processing, is through application of pressure, reducing the milk fat globule size and preventing fat separation from the milk. Other treatments that cause the same homogenization effect with pressure in milk are ultra high pressure, ultrasound, microfluidization and pulsed electric fields. Each type of homogenization causes additional effects on milk and on the produced yogurt. Finally, conventional fermentation process, in yogurt manufacturing, included the utilization of the species *Streptococcus salivarius* subsp. *thermophilus* and *Lactobacillus delbrueckii* subsp. *bulgaricus*. Modern dairy science and nutrition have suggested the involvement of probiotic cultures and prebiotic ingredients in order to increase the nutritional value of dairy products, while minimizing detrimental effects on the sensory characteristics.
